# Ultrastable Water‐in‐Water Emulsions by Addition of Polyion Complex Micelles

**DOI:** 10.1002/marc.70397

**Published:** 2026-08-28

**Authors:** Léna S. Vincent, Vendini Poopalan, Mariam Koite, Mohamed Hanafi, Jean‐Michel Guigner, Ilja K. Voets, Patrick Perrin, Cécile Monteux, Théo Merland

**Affiliations:** ^1^ Soft Matter Sciences and Engineering ESPCI PSL University Sorbonne Université Paris France; ^2^ Institut de Minéralogie Physique des Matériaux et de Cosmochimie Sorbonne Université Paris France; ^3^ Laboratory of Self‐Organizing Soft Matter Department of Chemical Engineering and Chemistry & Institute for Complex Molecular Systems Eindhoven University of Technology Eindhoven Netherlands

**Keywords:** confocal microscopy, light scattering, long term stability, polyion complex micelles, salt‐responsiveness, water‐in‐water emulsions

## Abstract

We report the use of polyion complex micelles (PICM) obtained by complexation of poly(sodium acrylate) and poly(N‐methyl‐2‐vinylpyridinium iodide‐*block*‐ethylene oxide) as novel stabilizers for water‐in‐water emulsions consisting in poly(ethylene oxide) (PEO) and dextran mixtures in water. PICM concentrations as low as 1 g/L lead to a strong increase of stability for both PEO‐in‐dextran and dextran‐in‐PEO emulsions, as they did not display any macroscopic phase separation after several months. Such long term stability is to our knowledge unprecedented in the literature dedicated to water‐in‐water emulsions. Moreover, increasing the concentration of PICM further induces a decrease in the droplet size, resulting in a strong reduction of the cream‐ing or sedimentation rates, and suppression of the coalescence after a few days. The dissociation of PICM at high salt concentration was taken as an opportunity to trigger the destabilization of the water‐in‐water emulsions by addition of a concentrated salt solution. PEO‐in‐dextran emulsions displayed more sensitivity to PICM and salt concentrations than dextran‐in‐PEO emulsions, in terms of both stability and droplet size, indicating that the underlying stabilization mechanisms are different for the two systems.

## Graphical abstract

Water‐in‐water emulsions from mixtures of dextran and poly(ethylene oxide) (PEO) prepared in the presence of polyion complex micelles display an increase of stability from few hours to several months. Both dextran‐in‐PEO and PEO‐in‐dextran were stable against coalescence over this period of time. Their destabilization was triggered within a few hours by adding a concentrated salt solution that dissociated the micelles.

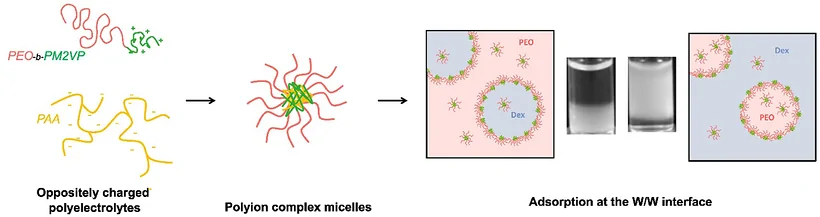

**Abbreviations**
PICM: polyion complex micelle
W/W: water‐in‐water
D/P: dextran‐in‐poly(ethylene oxide)
P/D: poly(ethylene oxide)‐in‐dextran
M_app_
: apparent molar mass
PM2VP‐*b*‐PEO: poly(N‐methyl‐2‐vinylpyridinium iodide‐*block*‐ethylene oxide)
PAA: poly(sodium acrylate)
PEO: poly(ethylene oxide)
R_g_
: radius of gyration
R_h_
: hydrodynamic radius

Water‐in‐water (W/W) emulsions, formed by segregative phase separation of two incompatible aqueous polymer solutions, [1, 2, 3] have attracted increasing attention for texturation [4, 5, 6] or as biocompatible templates for encapsulation, controlled release, and compartmentalization [7, 8, 9, 10, 11, 12]. For such applications, W/W emulsions present advantages with respect to oil/water emulsions as they avoid the use of fat for food or cosmetic formulations or toxic organic solvents as the organic phase. However, the practical use of W/W emulsions is strongly limited by their intrinsic lack of stability. Indeed, W/W emulsions cannot be stabilized by surfactants as they are fully hydrophilic, their interface is much larger than that of oil/water systems (several nanometers vs few ångström) and their interfacial tension is much lower (ca. 10^−3^–10^−1^ mN/m vs 1–50 mN/m), which prevents the adsorption of small molecules at the interface. Over the last decade, numerous studies have demonstrated that colloidal particles can adsorb at the W/W interface and form a barrier to droplet coalescence, leading to so‐called Pickering W/W emulsions [13, 14, 15, 16, 17]. This phenomenon requires the use of particles of sufficiently large size to increase significantly the desorption energy, as well as having an affinity of the particle for both phases, sometimes referred to as bis‐hydrophilicity [18, 19, 20, 21]. Moreover, it has been observed that particles that strongly interact at the interface better prevent the coalescence of the droplets [19]. The irreversible adsorption of particles at the water‐in‐water interface has recently been used to develop colloidosomes [17, 22]. Beyond particles, previous studies have also reported that homopolymers [23] and diblock [24, 25, 26] or triblock [27] copolymers can stabilize W/W emulsions. Again, the bis‐hydrophilic nature of the stabilizing polymers used in these systems may play a central role, as it provides an intrinsic driving force for their partitioning in the emulsions and their conformation at the interface. However, for both polymers and particles, the literature lacks comprehensive studies on the relationship between their chemical composition and their ability to stabilize the W/W interface, by adjusting their bis‐hydrophilicity and their interactions at the interface.

In this context, polyion complex micelles (PICM) [11, 28, 29], also known as complex coacervate core micelles (C3M) [30, 31, 32], polyelectrolyte complex micelles (PCM) [33, 34], or (inter)polyelectrolyte complex (PEC/IPEC) micelles [35, 36], offer an attractive and versatile class of hydrophilic soft colloidal stabilizers. They are formed through electrostatic interaction between two oppositely charged polyelectrolytes, to which a neutral hydrophilic block is attached. Their co‐assemblies display a core‐corona structure, with a dense polyelectrolyte‐rich core surrounded by a hydrated corona of neutral polymer chains [11]. Their radius and morphology can be tuned by varying the ratio between the neutral and charged blocks, as well as the block lengths [37, 38, 39, 40, 41]. In the present work, we describe the stabilization of W/W emulsions composed of poly(ethylene oxide) (PEO) and dextran by PICM with a PEO corona. We hypothesize that the corona should have a higher affinity with the PEO phase, so their core should prefer dextran in order to achieve bis‐hydrophilicity, and hence, stabilization of PEO/dextran emulsions (see Scheme 1).

**SCHEME 1:**
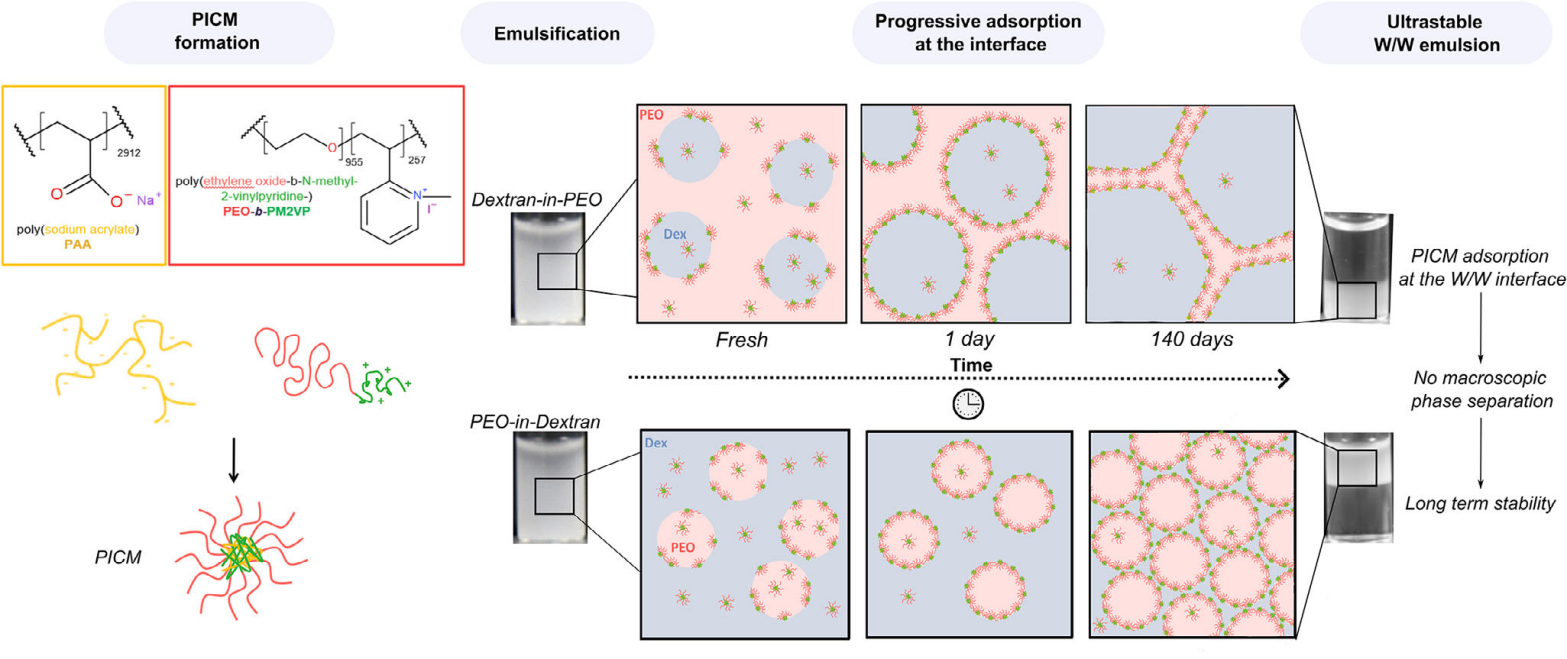
Schematic representation of PICM formation by complexation of PAA and PM2VP‐*b*‐PEO and their progressive adsorption at the dextran/PEO interface for D/P emulsions (top) and P/D emulsions (bottom). Note that the scheme is not to scale as PICM are two orders of magnitude smaller than droplets.

The PICM used in this work were obtained by mixing a poly(sodium acrylate) homopolymer (PAA) and a poly(N‐methyl‐2‐vinylpyridinium iodide‐*block*‐ethylene oxide) diblock copolymer (PM2VP‐*b*‐PEO) (see Scheme 1). The complexation of the two polymers led to the occurrence of a slight opalescence in the mixture (Figure 1a) and was confirmed by light scattering measurements in dilute conditions (Figure 1b,c). Indeed, the mixture displayed a higher scattering intensity than the sum of the two separate polyelectrolyte solutions, indicating attractive interactions [11, 23]. Light scattering experiments did not display any concentration dependence between 0.25 and 1 g/L (Figure 1c). The radius of gyration (R_g_) was equal to 30 nm and about a factor 2 lower than the hydrodynamic radius (R_h_). The PICM have a molar mass equal to 3.5±0.4 × 10^6^ g/mol. Making the hypothesis that all polymer chains are involved in the complexes, we can estimate the number of chains in a PICM from the charge ratio, the number of charged units in each polyelectrolyte, and their molar masses. In this way, the average composition of a PICM was found to be comprising 1.4 PAA chain and 18.1 PM2VP‐*b*‐PEO chains (see detailed calculation in Supporting information). With cryo‐transmission electron microscopy (cryoTEM), the dense cores of the micelles were observed (Figure 1d), but the contrast of the PEO corona was too low to observe them because of their high hydration and the low electron density of PEO, as also reported by Voets et al. [42]. The cores are spherical with a mean radius of 12±2 nm, and their random distribution across the sample confirms that there is no lang range organization at 1 g/L.

**FIGURE 1:**
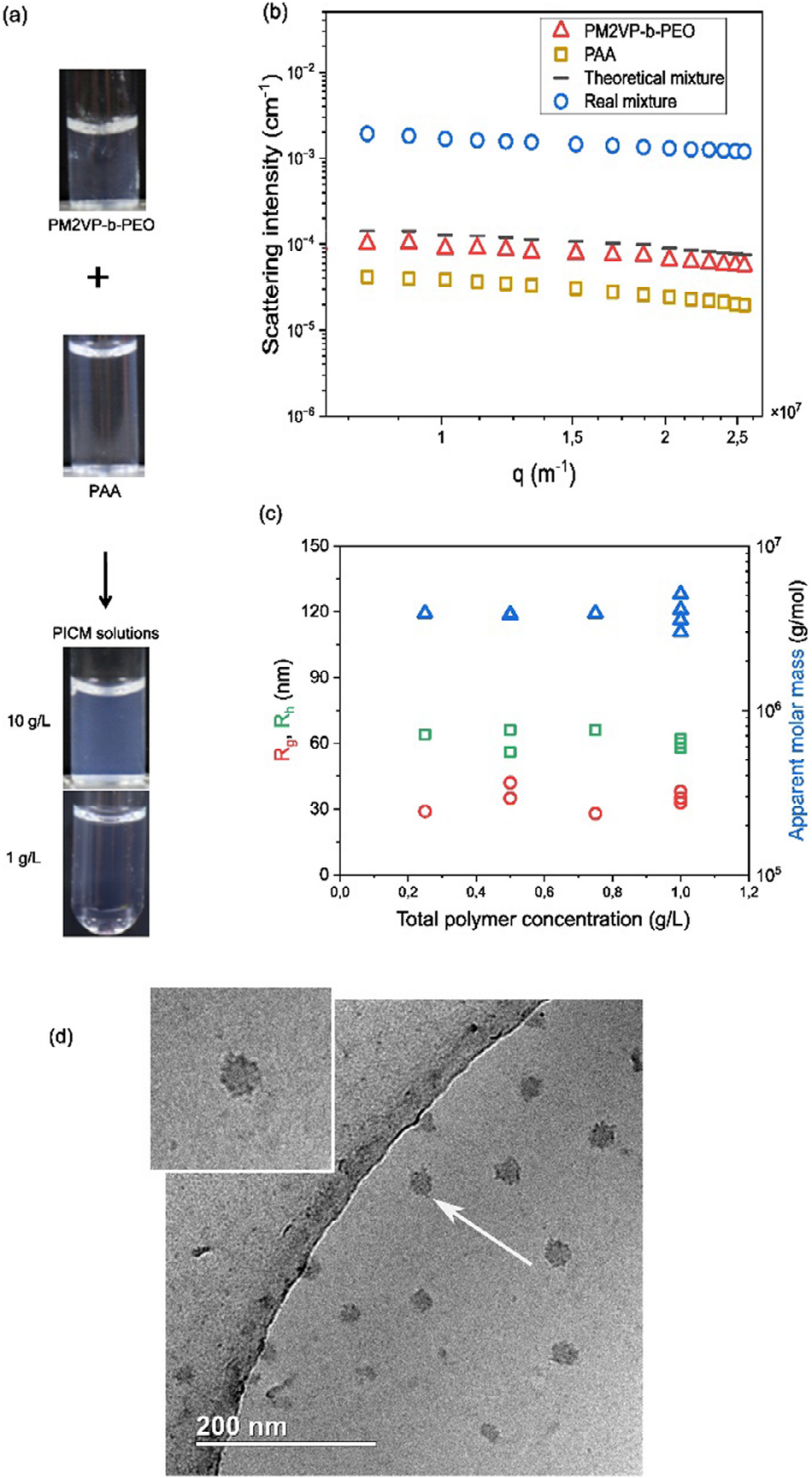
(a) Photographs of aqueous solutions of aqueous solutions of PM2VP_257_‐*b*‐PEO_955_ (8.5 g/L), PAA_2913_ (1.5 g/L), and their mixture (10 and 1 g/L). See Supporting Information for more information regarding the preparation of PICM. (b) Scattering intensity, expressed as the Rayleigh ratio, as a function of the scattering wave vector for aqueous solutions of PM2VP_257_‐*b*‐PEO_955_ (0.85 g/L), PAA_2913_ (0.15 g/L), their theoretical mixture equal to the sum of their scattering intensities and their real mixture (1 g/L) at pH 11. (c) Radius of gyration (red circles) and hydrodynamic radius (green squares) of PICM on the left axis and apparent molar mass (blue triangles) on the right axis, as a function of the total polymer concentration. Detailed light scattering data along with the method for determining M_app_, R_g_ and R_h_ are given in Supporting Information (Figure S4). (d) Cryo‐transmission electron microscopy image of a 1 g/L solution of PICM. The white arrow points out to the core of the micelles. The insert is an enlarged view of the image. More images are available in Figure S13.

PEO and dextran were used to formulate model W/W emulsions with 25 vol% dispersed phase and a relatively high interfacial tension of 75 µN/m [18]. The PEO phase contained 8 wt.% polymer and the dextran phase 16 wt.%. Depending on the composition, either PEO droplets in a continuous dextran phase (P/D) or dextran droplets in a continuous PEO phase (D/P) were obtained. The photographic images of the aging dynamics of P/D and D/P emulsions containing 1 g/L of PICM is shown in Figure 2a. Both types of emulsions displayed mild turbidity at the initial stage after vortex shaking. First, the control D/P emulsion (i.e. without PICM) displayed full macroscopic phase separation after a day. In contrast, in the presence of PICM, a partial sedimentation of the droplets in the D/P emulsion was observed after 1 day, due to the higher density of the dextran phase, resulting in a clarification of the upper part of the samples observed after 1 day. However, no macroscopic phase separation of the dextran phase occurred. A steady state was reached over one week when the volume fraction of the turbid part of the sample remained constant. Even after 20 weeks, the emulsion remained stable against coalescence, demonstrating the long term protective effect of PICM.

**FIGURE 2:**
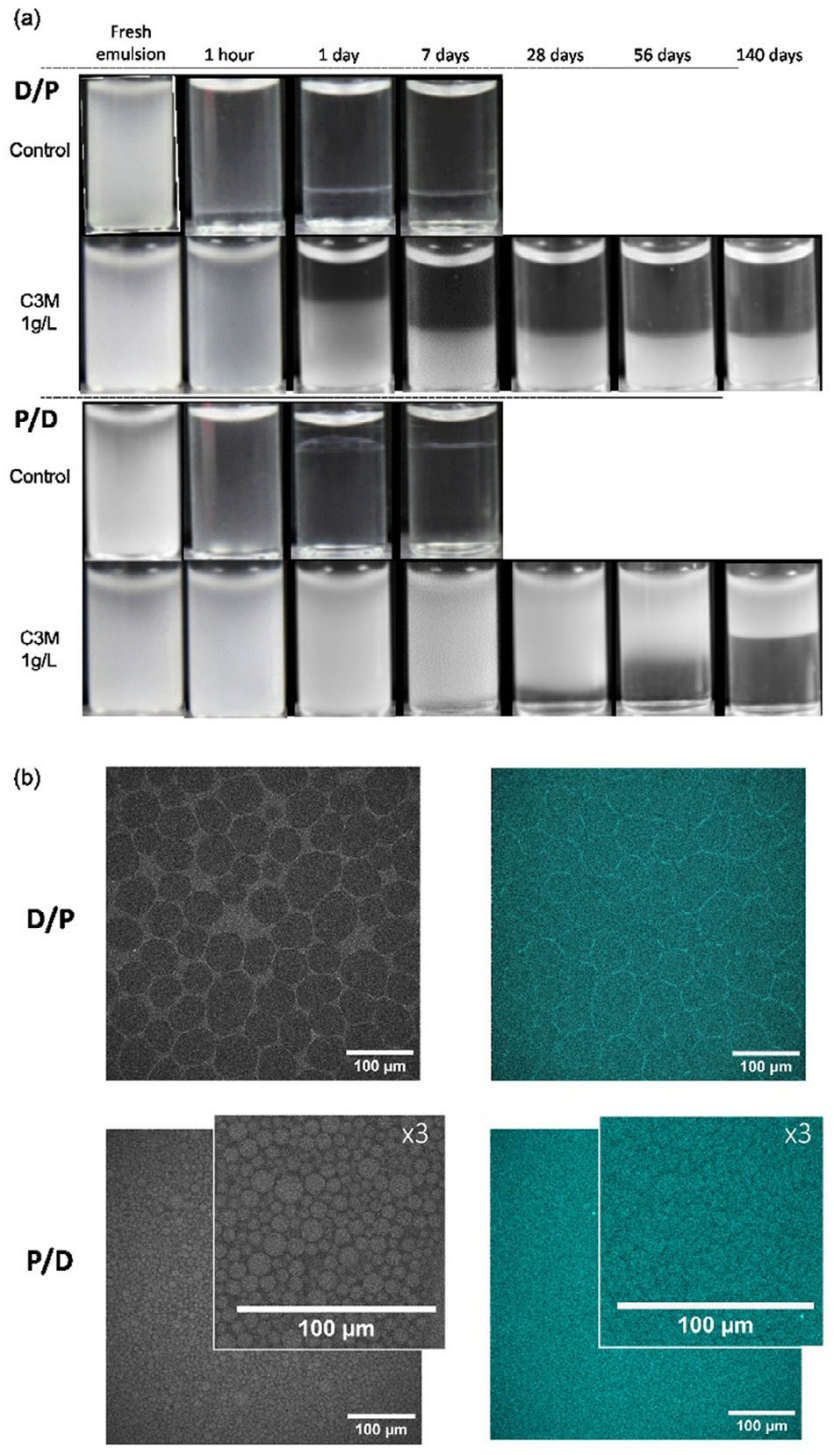
(a) Photographs of D/P (top) and P/D (bottom) emulsions containing 0 and 1 g/L of PICM at different aging times. (b) CLSM images the emulsified domain of D/P and P/D emulsions stabilized with 1 g/L of PICM, after aging for one week. Grey color (left) refers to the presence of rhodamine B (0.1 ppm), that was added to the emulsions and partitioned preferentially into the PEO phase. Cyan color (right) refers to PICM that are formed using fluoresceinamine‐grafted PAA. Additional images are provided at shorter times in Figures S7 and S8.

For P/D emulsions, while the control sample was also destabilized after a few hours, the presence of PICM leads to a strong delay of the creaming process which is observed after approximately one week, as evidenced by the clarification of the lower part of the tube. A steady state was reached after around 20 weeks. No macroscopic phase separation was observed over the whole study for both types of emulsions.

The emulsified part of the samples was therefore investigated with confocal laser scanning microscopy (CLSM), as shown in Figure 2b. In P/D emulsions, small droplets with a relatively narrow distribution were evidenced, with a mean radius of 4.0 ± 1.8 µm, consistent with the slow droplet migration rate at the macroscopic scale (see Figure 2b).

No significant evolution in the droplet size or dispersity was detected during aging of P/D (see Figure S7). In contrast, the dextran droplets in PEO increased in size during aging (Figure S8), and after reaching the steady state, they packed at the bottom of the sample (Figure 2b). Microscopic investigation of D/P emulsions using fluorescently labeled PAA to form PICM further revealed a progressive accumulation of PICM at the interface after one day. The absence of fluorophore at the edge of the droplets of fresh D/P emulsions suggests that interfacial adsorption develops over time. For P/D emulsions, no preferential partitioning of PICM at the interface was observed, even after one week. Individual PICM were not observed because their size is lower than the resolution of CLSM. It is worth mentioning that emulsions prepared with the same concentration of fluoresceinamine‐grafted PAA in the absence of PM2VP‐*b*‐PEO displayed accumulation of PAA at the interface of fresh D/P emulsions, but the emulsions were not significantly more stable than in the absence of PAA (Figure S9). In the case of P/D emulsions, PAA did not partition preferentially at the interface and nor did it stabilize the emulsions.

Altogether, these observations confirm that PICM effectively arrest coalescence in both emulsion types, but also reveal distinct adsorption dynamics and interfacial organizations in D/P and P/D emulsions. This naturally raises the question of the underlying stabilization mechanism. Es Sayed and coworkers have recently reported the use of PICM as efficient oil/water emulsions Pickering stabilizers, confirming their ability to adsorb at fluid–fluid interfaces and protect the droplets from coalescence [43]. However, in this example, the corona itself is composed of poly(N‐isopropylacrylamide) which is well known for its interfacial activity, and the core plays the role of anchoring point for the surface‐active chains. In the case of W/W emulsions, the stabilization of the interface by polymers remains unclear. Some works have proposed micelle‐driven adsorption [24, 44, 45], while others suggest the formation of a continuous polymer layer at the interface [23, 27, 46, 47, 48]. Here, the PEO corona of PICM is hypothesized to preferentially interact with the PEO phase. From this assumption and the observation that PICM stabilize both D/P and P/D emulsions, we can conclude that the core of the PICM has a higher affinity for the dextran phase. At the PEO/dextran interface, we suggest that the particles re‐arrange in a jellyfish‐like conformation so that the core is located in the dextran phase and the corona protrudes in the PEO phase (Scheme 1). Further investigation using Small‐Angle x‐Ray Scattering to elucidate the structure at the interface will be the subject of a future study. It is worth noting that in D/P systems, the packing after sedimentation deformed the droplets without inducing further coalescence (Figure 2b), contrary to creamed P/D emulsions (Figure S12). We suggest that the high deformability of the interface is due to weak interparticle interactions and/or decrease of interfacial tension.

As shown in Figure 3a, the stability of both P/D and D/P emulsions strongly depends on PICM concentration. For D/P emulsions, full sedimentation was completed within a week at low PICM concentrations, while at 2 g/L, sedimentation was still ongoing after a week. In all D/P samples containing 1 g/L PICM or more, a persistent turbidity remained at the bottom of the tube, indicating that the emulsions were protected against coalescence. For P/D emulsions, creaming was still ongoing after 70 days for 1 and 2 g/L, whereas the steady state was reached after about 35 days for 0.5 g/L. Overall, the creaming velocity decreased with increasing PICM concentration. To quantify the effect of the latter on emulsion stabilization, the evolution of droplet size over time was monitored by confocal microscopy (Figure 3b,c). In both D/P and P/D emulsions, the droplet size decreases with increasing PICM concentration, in line with the slower creaming or sedimentation observed macroscopically. At 2 g/L of PICM, droplets in D/P and P/D reach similar sizes (Figure 3b). Therefore, the much faster sedimentation of the D/P emulsion compared to the creaming in the P/D emulsion can only be explained by flocculation of the droplets in the first. Indeed, microscopic investigation of the emulsions one day after dispersion showcased aggregation of the dextran droplets (Figure S11), whereas no flocculation was detected for P/D emulsions. Interestingly, the enhancement of stability was observed only above a critical concentration of PICM that is higher for D/P (1 g/L) than for P/D (0.5 g/L).

**FIGURE 3:**
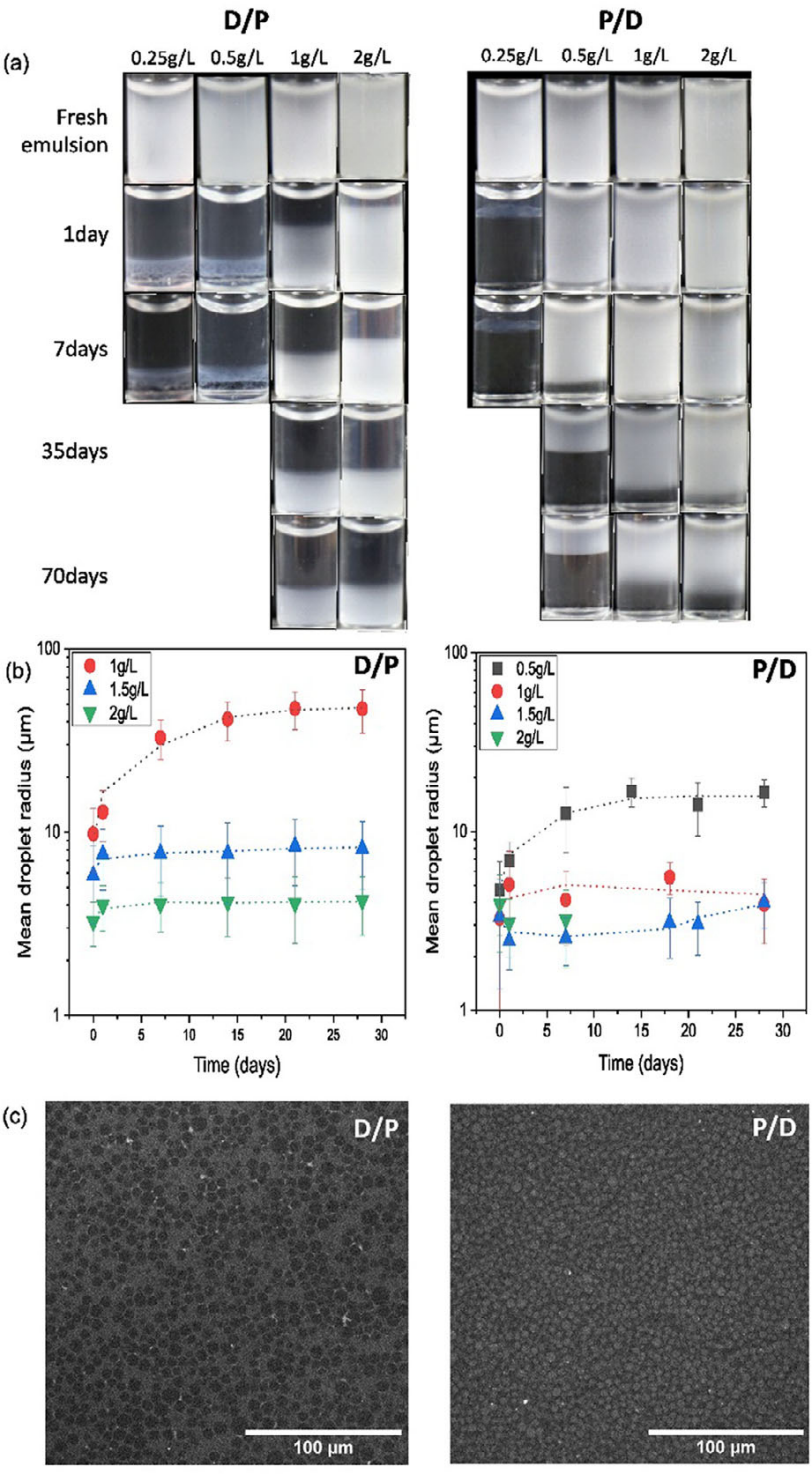
(a) Photographs of D/P (left) and P/D (right) emulsions containing various concentrations of PICM at various aging times. (b) Impact of concentration of PICM on the mean droplet radius of D/P (left) and P/D (right) emulsions over time. Error bars represent the standard deviation of the measured droplet radii. Detailed droplet size distributions of fresh and 28‐days‐old W/W emulsions are given in Figure S10. (c) CLSM images of the emulsified domain of 7‐day‐old D/P (left) and P/D (right) emulsions stabilized with 2 g/L of PICM. Grey color refers to the presence of rhodamine B (0.1 ppm), that was added to the emulsions and partitioned preferentially into the PEO phase.

This may arise from a difference in PICM adsorption kinetics and interfacial coverage in the two cases, consistent with the progressive accumulation of fluorescence at the interface previously observed for D/P emulsions. Just above these critical concentrations, droplet growth occurred during several days before reaching a plateau. This behavior suggests that initial coalescence occurs due to insufficient interfacial coverage, until the interface is fully covered by PICM so that coalescence is prevented. When PICM concentration is much above this critical value, in both cases the droplet size did not vary much after a day.

Remarkably, very few stabilizers were reported to provide comparable protection against coalescence, especially at such low concentrations. To the best of our knowledge, one of the most efficient stabilizers for PEO/dextran systems was reported by González‐Jordan et al., who showed that 0.5–1 g/L of β‐lactoglobulin fractal aggregates and microgels efficiently limit coalescence and significantly slow creaming or sedimentation in either P/D or D/P emulsions [15]. In another study, Waldmann et al. reported that 0.5 g/L poly(N‐isopropylacrylamide)‐co‐dextran microgels can delay macroscopic phase separation of P/D and D/P systems, although only when the dextran mass fraction in the microgels is sufficiently high [21]. Moreover, these two studies did not report stability over extended periods at the scale of several months as in the present study.

PICM can be dissociated by addition of an excess of salt [28]. This sensitivity to salt can thus be exploited as a stimulus to trigger on demand the destabilization of PICM‐stabilized W/W emulsions. Remarkably, a P/D emulsion containing 1 g/L of PICM—stable for months in the absence of added salt as demonstrated above—underwent phase separation within 24 h after increasing added NaCl concentration from 0 to 0.2 м by addition of a concentrated salt solution (Figure 4a). Indeed, Figure 4b shows that at a NaCl concentration of 0.2 м, the sum of the scattering intensities of the individual polymers was equal to that of the PICM stock solution, indicating that no attractive interactions took place between the two polyelectrolytes. The dissociation of PICM in the presence of 0.2 м added NaCl was also observed by cryoTEM imaging (Figure S13d). This is because added salt screens the attractive electrostatic interactions in the PICM core. As a result, beyond a critical salt concentration, the micelles are dissociated [29, 37]. Also, the diblock copolymer exhibited a slight aggregation (Figure S13b) [48]. Moreover, fresh P/D emulsions prepared with 0.2м NaCl rapidly phase separated (Figure 4c): most droplets creamed within 2 h, and the appearance of a clear upper phase confirms that coalescence has occurred. For D/P emulsions, the phase separation was slower and occurred within few days. The higher stability of D/P emulsions at 0.2 м NaCl could be due to the adsorption of aggregates of PM2VP‐*b*‐PEO at the interface [49], although the emulsions remain significantly less stable than in pure water. The loss of stabilizing efficiency arises from the salt‐induced dissociation of PICM. Consequently, in W/W emulsions, the interface is no longer effectively protected, leading to emulsion destabilization (Figure 4d).

**FIGURE 4:**
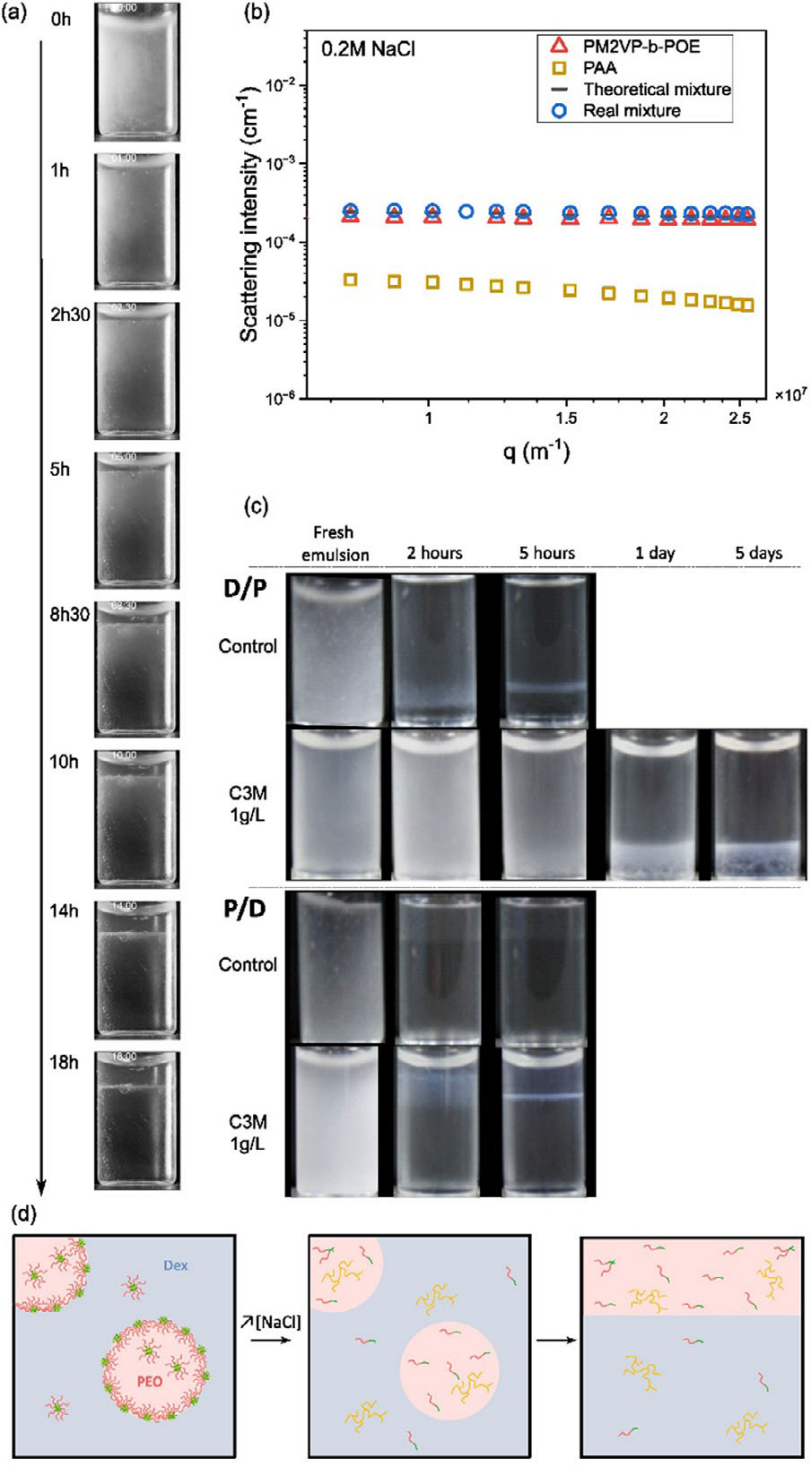
Effect of salt. (a) Time‐lapse images (from a video available in the Supporting Information) showing the destabilization of a 1 g/L PICM‐stabilized P/D emulsion upon addition of NaCl (final concentration 0.2 м). (b) Scattering intensity, expressed as the Rayleigh ratio, as a function of the scattering wave vector of aqueous solutions of PM2VP_257_‐*b*‐PEO_955_ (0.85 g/L), PAA_2913_ (0.15 g/L), the mixture (1 g of polymers per L) adjusted at pH 11, in presence of 0.2м NaCl. (c) Photographs of D/P (top) and P/D (bottom) emulsions containing 0 g/L (control) and 1 g/L of PICM prepared at 0.2 м NaCl. (d) Schematic representation of on‐demand destabilization of a P/D emulsion: salt‐induced PICM dissociation exposes the interface leading to a macroscopic phase separation.

In conclusion, we have shown that PICM are excellent stabilizers for both P/D and D/P emulsions. In both cases, only 1 g/L PICM are required to stabilize water‐in‐water emulsions for at least five months. A relationship between droplet size and the concentration of PICM was established showing that coalescence is clearly suppressed and droplet size remains constant over months. The long term stability evidenced in our study is unprecedented in the literature for W/W emulsions. Moreover, W/W emulsions stabilized by PICM can be destabilized on demand by increasing the ionic strength above the PICM dissociation critical value, providing a convenient way to dynamically control emulsion stability. The mechanisms of stabilization are not the same for D/P and P/D, because their microstructure was very different and they did not display the same sensitivity to PICM and salt concentrations. We suggest that the key for the comprehension of this system lies in the interactions between the particles and their surrounding medium, which we are currently investigating and will report in a near future.

The adsorption behavior of PICM and their ability to form effective interfacial barriers highlight a new route for stabilizing W/W emulsions with stimuli‐responsive, soft colloids. The high versatility of PICM which composition can be varied to tune their affinity for each phase, as well as their size and morphology [30, 32, 37, 50], paves the way toward understanding and thus, optimizing their stabilizing effect, by tailoring them for each W/W system of interest. Beyond their stability, these systems can display both segregative and associative liquid‐liquid phase separations, the first being the droplets at the micrometric scale and the second being the core of the micelles at the nanometric scale. This feature opens up perspectives as for their ability to encapsulate two types of species in different types of compartments, to achieve complex chemical systems for catalysis or cascade reactions [51]. Achieving such hierarchically compartmentalized systems would be useful for the design of complex colloids such as synthetic cells in which membraneless organelles are typically replicated using segregative phase separation [52, 53].

Additional references were cited in the Supporting Information [54, 55, 56].

## Conflicts of Interest

The authors declare no conflicts of interest.

## Supporting information




**Supporting File 1**: marc70397‐sup‐0001‐SuppMat.docx.

**Supporting File 2**: marc70397‐sup‐0002‐Video 1.avi.

## Data Availability

The data that support the findings of this study are available from the corresponding author upon reasonable request.
