# Supplementary material for: Ultrastable Water‐in‐Water Emulsions by Addition of Polyion Complex Micelles
Source: Macromol Rapid Commun. 2026 Aug 28;47(19):e70397. doi: 10.1002/marc.70397 (PMC13639201; doi:10.1002/marc.70397)
Supplement: Supplementary file 1 [file MARC-47-e70397-s002.docx]

**Supporting Information for**

Ultrastable water-in-water emulsions by addition of polyion complex micelles

Léna S. Vincent,^1^ Vendini Poopalan,^1^ Mariam Koite,^1^ Mohamed Hanafi,^1^ Jean-Michel Guigner,^2^ Ilja K. Voets,^3^ Patrick Perrin,^1^ Cécile Monteux,^1^ and Théo Merland^*,1^

^1^Soft Matter Sciences and Engineering, ESPCI, PSL University, Sorbonne Université, CNRS UMR 7615, 10 rue Vauquelin, 75231 Paris Cedex 05, France

^2^*Institut de Minéralogie, Physique des Matériaux et de Cosmochimie, Sorbonne Université, CNRS UMR 7590, MNHN, F-75005 Paris, France*

*^3^Laboratory of Self-Organizing Soft Matter, Department of Chemical Engineering and Chemistry & Institute for Complex Molecular Systems, Eindhoven University of Technology, P.O. Box 513, 5600, MB Eindhoven, the Netherlands*

Contents

[Materials.	2](#_Toc227947138)

[pH titration and characterization of PAA.	2](#_Toc227947139)

[Quaternization of poly(2-vinylpyridine) and Nuclear Magnetic Resonance.	5](#_Toc227947140)

[Preparation of PICM and characterization by light scattering.	6](#_Toc227947141)

[Preparation and characterization of water-in-water emulsions.	8](#_Toc227947142)

[Fluorophore grafting and CLSM imaging.	10](#_Toc227947143)

[Cryo-TEM observations.	15](#_Toc227947144)

[Size-exclusion chromatography.	16](#_Toc227947145)

[References	17](#_Toc227947146)

# Materials.

Poly(ethylene oxide) (PEO, M_v_= 200 kg/mol), dextran (M_r_=450-650 kg/mol), poly(acrylic acid) (PAA, M_v_= 450 kg/mol), rhodamine B, and 6-aminofluorescein were purchased from Sigma-Aldrich. The polymers were characterized using size exclusion chromatography (see Figure S7). Poly(2-vinylpyridine)-*block*-PEO (P2VP-*b*-PEO) (M_n_=27-*b*-42 kg/mol) was purchased from Polymer Source (Dorvel, Canada).

# pH titration and characterization of PAA.

To determine the ionization degree of PAA as a function of pH, a titration on a 1.6 g/L PAA solution was performed using 0.1 M NaOH as titrant. Prior to the titration, 1 mL of 0.1 M HCl was added to the solution to ensure that the system started from a well-defined acidic state. The transition region, corresponding to the progressive deprotonation of the carboxyl groups, was used to identify the apparent pKa of the polymer. The midpoint of the titration curve was found at pH ≈ 9, which was taken as the apparent pKa. It can be used to calculate the degree of ionization with the following equation.^42^

$$\alpha=\frac{{10}^{pH-pKa}}{1+{10}^{pH-pKa}}$$

**Figure S1.** (a) Titration curve of PAA. The titration volume includes the NaOH required to neutralize the HCl added prior to the titration. (b) Ionization degree of PAA as a function of pH.

**(a)**

| Wavenumber (cm^-1^) | Assignment | Vibration mode |
| --- | --- | --- |
| 3050 | O-H | stretching |
| 2950 | C-H | stretching |
| 1705 | C=O | stretching |
| 1455 | C-H (CH_2_) | bending |
| 1415 | O-H | bending |
| 1250 | C-O | stretching |
| 920 | O-H | bending |

**(b)**

**(c)**

**Figure S2.** (a) ATR-FTIR spectrum of PAA with peak attribution. (b) ^1^H and (c) ^13^C NMR spectra of PAA in D₂O. Peaks labeled with a * are signals from the solvents.

# Quaternization of poly(2-vinylpyridine) and Nuclear Magnetic Resonance.

P2VP-*b*-PEO was quaternized with methyl iodide according to the procedure described by Voets *et al.*^54^ Briefly, the polymer was dissolved in DMF at room temperature and degased with N_2_. A degased solution of methyl iodide in DMF was then added dropwise and the mixture was heated to 60°C for 48h. It was then precipitated and washed in diethyl ether, yielding a light yellow powder.

^1^H NMR experiments in D_2_O and CDCl_3_ were performed on a Bruker AV Neo 400 MHz spectrometer at 23°C. Before quaternization, the P2VP-*b*-PEO copolymer was poorly soluble in water, which required NMR measurements to be carried out in CDCl₃. After quaternization, the resulting PM2VP-*b*-PEO became water-soluble, and noticeable changes were observed in the ^1^H NMR spectra. In particular, the spectra displayed larger signals, with the most significant difference being the integration of the PEO signal around 3.6 ppm. A previous study assigned a new signal at 4.2 ppm, to the methyl group of a quaternized poly(4-vinylpyridine) homopolymer.^55^ Although no distinct new peak is observed here for PM2VP, the increase in integration can be attributed to the introduced methyl group. Based on this assignment, the quaternization degree was calculated using the following equation (see below), with a value of 54%.

$$Quaternization degree (Q)= \frac{I\left( POE \right)_{P2MVP}-I\left( POE \right)_{P2VP}}{I(CH_{3})}$$

**(a)**

**(b)**

**Figure S3.** ^1^H NMR spectra of P2VP-*b*-PEO (a) in CDCl_3_ before quaternization and (b) in D_2_O after quaternization. Peaks labeled with a * are signals from the solvents.

# Preparation of PICM and characterization by light scattering.

The polyelectrolytes were dissolved in water and pH was adjusted to 11 by addition of aliquots of NaOH to ensure full ionization of PAA (see Figure S1). Then, the PM2VP-*b*-PEO solution was placed under vigorous stirring, and the PAA solution was added dropwise. The resulting PICM were characterized by static and dynamic light scattering at 20°C using an ALV/CGS-3 compact goniometer equipped with a λ=633 nm He-Ne laser, at angles varied between 12 and 150° and an ALV-5001 correlator.

The hypothesis that all polymer chains were involved in PICM was made. The weight fraction of each polyelectrolytes in the PICM was computed from this assumption and considering that all PAA repeating units are ionized at pH 11 as shown above, and that 54% of the P2VP repeating units were quaternized and thus, converted in PM2VP units in the cationic block copolymer.

We found $\omega_{PAA}=0.15$ and $\omega_{P2MVP-b-PEO}=0.85$.

The contrast of PICM is therefore $\frac{dn}{dC}=\omega_{PM2VP-b-PEO}\left( \frac{dn}{dC} \right)_{P2MVP-b-PEO}+\omega_{PAA}\left( \frac{dn}{dC} \right)_{PAA}=0.201 mL/g$, using dn/dC equal to 0.231 and 0.195 respectively for PAA and PMVP-*b*-PEO, assuming the contrast is the same for PM2VP than for P2VP before quaternization.

PAA and P2MVP-b-PEO were mixed with a molar fraction of positive charges $f_{+}=\frac{n_{+}}{n_{+}+n_{-}}=0.39$ and their molar ratio is $R=\frac{n_{+}}{n_{-}}=\frac{N_{P2MVP-b-PEO}^{agg}\cdot x_{P2MVP-b-PEO}}{N_{PAA}^{agg}\cdot x_{PAA}}=0.64$ with N_i_^agg^ the number of polyelectrolyte i chains per PICM and x_i_ their number of charged units per chain. We thus found that $N_{P2MVP-b-PEO}^{agg}=13.4N_{PAA}^{agg}$.

From the weight average molar masses of PAA determined by SEC and that of PICM and PM2VP-*b*-PEO determined by static light scattering, we found that $N_{PAA}^{agg}=1.4$ and $N_{P2MVP-b-PEO}^{agg}=18.1$on average.

**Figure S4.** Static light scattering characterization of PICM prepared at various concentrations as indicated in the figure. The solid lines are fits of the data with the Zimm equation:

$$\frac{KC}{R_{\theta}}=\left( \frac{1}{M_{w}}+2A_{2}C+\ldots\right)\left( 1+\frac{q^{2}R_{g}^{2}}{3} \right)$$

$$K=\frac{4\pi^{2}n_{solv}^{2} (\frac{dn}{dc})^{2}}{\lambda^{4}N_{Av}}\times\frac{n_{tol}^{2}}{n_{solv}^{2}}$$

With M_w_ the apparent weight average molar mass, A_2_ the second virial coefficient, q the wavevector, R_g_ the radius of gyration, n_solv_ the refractive index of the solvent, n_tol_ the refractive index of toluene that was used as a standard, and N_av_ the Avogadro number.

# Preparation and characterization of water-in-water emulsions.

Aqueous stock solutions of PEO and dextran were prepared at 10 wt% and 20 wt%, respectively, using ultrapure water (Millipore, USA). Before use, the PEO solution was centrifuged at 15,000 × g overnight to remove residual silica particles. W/W emulsions were prepared by mixing these two stock solutions in various amounts to reach 25 vol% of dispersed phase. This was achieved by making a mixture of 6 wt% PEO and 4 wt% dextran to form a dextran-in-PEO emulsion, and 2 wt% PEO and 12 wt% dextran to form a PEO-in-dextran emulsion. Their pH was adjusted to 11 by addition of NaOH and the mixtures were emulsified using a vortex mixer. PICM were then added to the W/W emulsions, and the mixtures were vortexed again to ensure homogenous dispersion.

W/W emulsions were prepared by mixing the appropriate amounts of PEO and dextran stock solutions. Their pH was adjusted to 11 by addition of NaOH and the mixtures were emulsified using a vortex mixer. PICM were then added to the W/W emulsions, and the mixture were vortexed again to ensure homogenous dispersion.

|  |  |
| --- | --- |

**Figure S5.** Photographs of D/P (left) and P/D (right) emulsions containing 0.85 g/L PM2VP-b-PEO or 0.15 g/L PAA as indicated in the figure, at different ageing times.

**Figure S6.** Flow curves of the PEO (8 wt%) and dextran (16 wt%) phases at pH 11 and 20 °C.

# Fluorophore grafting and CLSM imaging.

For their microscopic observation, the W/W emulsions were placed between a concave slide and a cover slip separated by a 1 mm spacer. The images were obtained using a Leica DM8i confocal microscope (Leica Microsystems, Germany), with a water immersion objective: HCX PL FLUOTAR 25×/0.75 W. 0.1 ppm rhodamine B was added to the W/W emulsionss to selectively stain the PEO phase. All experiments were carried out at T=20°C. PAA was labeled to enable selective visualization of the PICM as follows.

PAA was labeled with 6-aminofluorescein following a protocol adapted from Wang *et al*.^56^ The reaction was carried out in NMP using DCC as coupling agent. After 24 h at 60 °C, the mixture was filtered to remove byproducts and unreacted compounds, neutralized with NaOH, and precipitated in acetone. The orange solid was washed, dried under vacuum, and, when soluble, purified by dialysis against water. The grafting degree was determined by UV-Vis spectroscopy to be 0.004 mol%.

Droplet radii were estimated from 2D confocal microscopy images by measuring the projected area of each droplet and calculating the equivalent spherical radius. To correct for the projection of a sphere onto a 2D plane, the measured radius was multiplied by a factor of 4/π, which accounts for the difference between the mean area of random 2D slices and the actual 3D sphere surface. Size distributions were obtained by measuring at least 100 droplets in each case.

**Figure S7.** Confocal microscopy images of a fresh (left), 1-day-old (middle) and 7-day-old (right) P/D emulsion stabilized with 1 g/L PICM with rhodamine B (grey) that partitions preferentially into the PEO phase and PICM (cyan) that are formed using a mixture of fluoresceinamine-grafted PAA and PM2VP-*b*-PEO.

**Figure S8.** Confocal microscopy images of a fresh (left), 1-day-old (middle) and 7-day-old (right) D/P emulsion stabilized with 1 g/L PICM with rhodamine B (grey) that partitions preferentially into the PEO phase and PICM (cyan) that are formed using a mixture of fluoresceinamine-grafted PAA and PM2VP-*b*-PEO.

**Figure S9.** (a) Photographs of P/D (top) and D/P (bottom) emulsions containing 0.15 g/L fluoresceinamine-grafted PAA, at different ageing times. (b) Confocal microscopy images of fresh P/D (left) and D/P (right) emulsions in the presence of 0.15 g/L fluoresceinamine-grafted PAA.

**P/D**

|  | Fresh emulsion | 28-days old |
| --- | --- | --- |
| 0.5 |  |  |
| 1 |  |  |
| 1.5 |  |  |
| 2 |  |  |

**D/P**

|  | Fresh emulsion | 28-days old |
| --- | --- | --- |
| 1 |  |  |
| 1.5 |  |  |
| 2 |  |  |

**Figure S10**. Droplet size distributions for P/D (top) and D/P (bottom) emulsions fresh and aged for 28 days, at various PICM concentrations as indicated in the figure.

**Figure S11**. Confocal microscopy images of 1-day-old D/P emulsions stabilized with 1 g/L (left) and 2 g/L (right) PICM in the presence of rhodamine B that partitions favorably in the PEO phase (grey).

**Figure S12**. Confocal microscopy images of a 28-day-old P/D emulsion stabilized with 0.5 g/L PICM in the presence of rhodamine B that partitions favorably in the PEO phase (grey).

# Cryo-TEM observations.

Cryo-TEM samples were prepared by depositing 5 µL of the solution onto Quantifoil® carbon grids, blotting the excess, and rapidly vitrifying the thin film in liquid ethane using a homemade mechanical plunger. The grids were then transferred to a Gatan 626 cryo-holder cooled with liquid nitrogen and imaged at –180 °C using a JEOL JEM2100 microscope (200 kV, LaB₆ source) equipped with a low-dose system (MDS, JEOL, Japan) and an Ultrascan 1000 CCD camera (2k × 2k, Gatan, USA).

**Figure S13**. Cryo-transmission electron microscopy image of a 8.5 g/L solution of PM2VP_257_-*b*-PEO_955_ without salt (a) and at 0.2 M NaCl (b), a 10 g/L solution of PICM (8.5 g/L PM2VP_257_-*b*-PEO_955_ + 1.5 g/L PAA_2913_) without salt (c) and at 0.2 M NaCl (d), and a 1.5 g/L solution of PAA_2913_ at 0.2 M NaCl (e). The carbon film appears darker and outlines the regular circular holes where the sample is vitrified. The short white arrows indicate polymers aggregates and the long white arrows indicate PICM. The black arrows indicate ice crystals.

# Size-exclusion chromatography.

Dextran, PAA, PEO were separately dissolved in an aqueous NaNO₃ solution (0.2 M) containing NaN₃ (0.02 wt%) at a concentration of 2 mg/mL, under stirring overnight to ensure complete solubilization. SEC measurements were carried out using a Viscotek SEC system equipped with three SHODEX OHpak SB-806M HQ columns (13 μm, 300 mm × 8 mm), thermostatically controlled at 35°C. The same NaNO₃/NaN₃ solution was used as the mobile phase, and samples were injected at a flow rate of 0.7 mL/min. All polymer solutions were filtered through 0.2 μm PVDF filters before measurements.

**Table S1**. Number-average molar mass (M_n_), weight-average molar mass (M_w_), and hydrodynamic radius (R_h_) obtained from SEC measurements.

|  | M_n_ (kg/mol) | M_w_ (kg/mol) | R_h_ (nm) |
| --- | --- | --- | --- |
| PEO | 62.8 | 215 | 16 |
| Dextran | 269 | 658 | 15 |
| PAA | 210 | 580 | 35 |
| Labelled PAA | 388 | 871 | 34 |

(c)

(b)

(a)

**Figure S14.** Size exclusion chromatograms of PAA (a), dextran (b), and PEO (c) in aqueous 0.2 M NaNO₃ solution (1 g/L).

# References

[42]	Es Sayed, J.; Brummer, H.; Stuart, M. C. A.; Sanson, N.; Perrin, P.; Kamperman, M. Responsive Pickering Emulsions Stabilized by Frozen Complex Coacervate Core Micelles. ACS Macro Lett. 2022, 11 (1), 20–25. https://doi.org/10.1021/acsmacrolett.1c00647.

[54]	Voets, I. K.; Fokkink, R.; de Keizer, A.; May, R. P.; de Waard, P.; Cohen Stuart, M. A. On the Transition between a Heterogeneous and Homogeneous Corona in Mixed Polymeric Micelles. Langmuir ACS J. Surf. Colloids 2008, 24 (21), 12221–12227. https://doi.org/10.1021/la801816p.

[55]	Mavronasou, K.; Zamboulis, A.; Klonos, P.; Kyritsis, A.; Bikiaris, D. N.; Papadakis, R.; Deligkiozi, I. Poly(Vinyl Pyridine) and Its Quaternized Derivatives: Understanding Their Solvation and Solid State Properties. Polymers 2022, 14 (4), 804. https://doi.org/10.3390/polym14040804.

[56]	Wang, K. T.; Iliopoulos, I.; Audebert, R. Viscometric Behaviour of Hydrophobically Modified Poly(Sodium Acrylate). Polym. Bull. 1988, 20 (6), 577–582. https://doi.org/10.1007/BF00263675.
